# The Mitochondrial Genome of *Toxocara canis*

**DOI:** 10.1371/journal.pntd.0000273

**Published:** 2008-08-06

**Authors:** Aaron R. Jex, Andrea Waeschenbach, D. Timothy J. Littlewood, Min Hu, Robin B. Gasser

**Affiliations:** 1 Department of Veterinary Science, The University of Melbourne, Werribee, Victoria, Australia; 2 Department of Zoology, The Natural History Museum, London, United Kingdom; University of South Florida, United States of America

## Abstract

*Toxocara canis* (Ascaridida: Nematoda), which parasitizes (at the adult stage) the small intestine of canids, can be transmitted to a range of other mammals, including humans, and can cause the disease toxocariasis. Despite its significance as a pathogen, the genetics, epidemiology and biology of this parasite remain poorly understood. In addition, the zoonotic potential of related species of *Toxocara*, such as *T. cati* and *T. malaysiensis*, is not well known. Mitochondrial DNA is known to provide genetic markers for investigations in these areas, but complete mitochondrial genomic data have been lacking for *T. canis* and its congeners. In the present study, the mitochondrial genome of *T. canis* was amplified by long-range polymerase chain reaction (long PCR) and sequenced using a primer-walking strategy. This circular mitochondrial genome was 14162 bp and contained 12 protein-coding, 22 transfer RNA, and 2 ribosomal RNA genes consistent for secernentean nematodes, including *Ascaris suum* and *Anisakis simplex* (Ascaridida). The mitochondrial genome of *T. canis* provides genetic markers for studies into the systematics, population genetics and epidemiology of this zoonotic parasite and its congeners. Such markers can now be used in prospecting for cryptic species and for exploring host specificity and zoonotic potential, thus underpinning the prevention and control of toxocariasis in humans and other hosts.

## Author Summary

*Toxocara canis* (Nematoda: Ascaridida) is the common roundworm of canids. This parasite is transmissible to humans as well as a range of other accidental or paratenic vertebrate hosts, in which (after the oral ingestion of infective eggs) the larvae of *Toxocara canis* invade the tissues and can cause different forms of clinical disease (toxocariasis). Although some aspects of the biology of *T. canis* are well understood, there are still significant gaps in our knowledge of areas including the molecular genetics, systematics, ecology and epidemiology of this and related parasitic nematodes. The present study elucidates the sequence, structure and organization of the mitochondrial genome of *T. canis* and provides mitochondrial gene markers for studies in these areas using molecular tools. A greater understanding of the epidemiology of species of *Toxocara* would improve the prevention and control of toxocariasis in humans and other animals.

## Introduction

Roundworms (nematodes) belong to a large phylum (Nematoda) in the animal kingdom. This phylum contains a wide range of species with exceptionally varied life histories [1]. Many nematodes are parasites of plants or animals [2], causing significant diseases and major socio-economic losses globally [3]–[5]. Central to the control of such parasites is knowledge of their population genetics, which has important implications for understanding many areas, including systematics, epidemiology and ecology [6]–[9]. The basis for investigating population structures is the accurate analysis of genetic variation, which is known to be widespread in many parasitic nematodes [6]–[8],[10], utilizing molecular markers with sufficient levels of intraspecific sequence variability.

Mitochondrial DNA markers are particularly suited to population genetic and systematic investigations due to their high mutation rates and proposed maternal inheritance [6], [7], [10]–[15]. In spite of the availability of advanced DNA technologies, there is still a paucity of knowledge of mitochondrial genomes for many parasitic nematodes of socio-economic importance [16], such as members of the Ascaridida, which is a key group of nematodes that parasitizes humans and a range of other vertebrates. Although complete mitochondrial genome sequences are available for *Anisakis simplex* [17] and *Ascaris suum* [18], this is not the case for other parasites within this order, such as *Toxocara canis*, the common roundworm of dogs. This latter nematode parasitizes (at the adult stage) the small intestine of canids (definitive host) and is also transmissible to a range of other mammals, including rodents and humans, in which (after the oral ingestion of infective eggs) the larvae of *Toxocara canis* invade the tissues and cause covert toxocariasis, ocular larva migrans (OLM), visceral larva migrans (VLM) or neurotoxocariasis [19]–[23]. Although there has been a significant acquisition of knowledge about the biology of *T. canis*, there are still major gaps in our knowledge of the genetics, ecology and epidemiology of this enigmatic parasite [24]. In addition, the detection of a cryptic species of “*T. canis*” from cats in Malaysia [25],[26], its subsequent genetic characterization [27] and its description as a new species - *Toxocara malayensis* [28], emphasize the need for detailed molecular genetic studies of *T. canis* populations using suitable genetic markers [29]. Exploring the mitochondrial genome of *T. canis* would provide such markers, as a foundation for molecular epidemiological and ecological studies, detecting cryptic species and assessing relationships of related species of *Toxocara* [29]. Furthermore, the sequencing of the mitochondrial genome of *T. canis* provides a useful, comparative dataset to those of *Anisakis simplex* [17] and *Ascaris suum* [18] within the order Ascaridida. Building on recent progress in long PCR-coupled, automated sequencing [30], the present study determined the sequence and structure of the mitochondrial genome for a representative individual of *T. canis* from Australia and compared it with those available for *Anisakis simplex* and *Ascaris suum* as well as the sequences from related nematode groups (Spirurida and Strongylida), as a foundation for systematic, population genetic and epidemiological studies of *T. canis*.

## Materials and Methods

An adult, male specimen of *Toxocara canis* (sample code Tcn2; ref. [31]) was collected (during a routine autopsy) from the small intestine of a fox from Victoria, Australia under the Scientific Procedures Premises License for the Faculty of Veterinary Science, University of Melbourne (SPPL045). Initially, the morphological identification of the worm was based on the presence of a post-oesophageal bulbus, the length and shape of the alae and the lengths of the spicules [32]. Total genomic DNA was extracted from a small portion (0.5 cm) of the specimen by sodium dodecyl-sulphate/proteinase K treatment, phenol/chloroform extraction and ethanol precipitation [33], and purified over a spin column (Wizard Clean-Up; Promega) [34]. In order to independently verify the identity of the specimen, the second internal transcribed spacer (ITS-2) of nuclear ribosomal DNA was amplified by the polymerase chain reaction (PCR) and sequenced according to an established method [27]. The ITS-2 sequence obtained was a perfect match with that of *T. canis* (accession number Y09489; ref. [34]).

**Figure 1:**
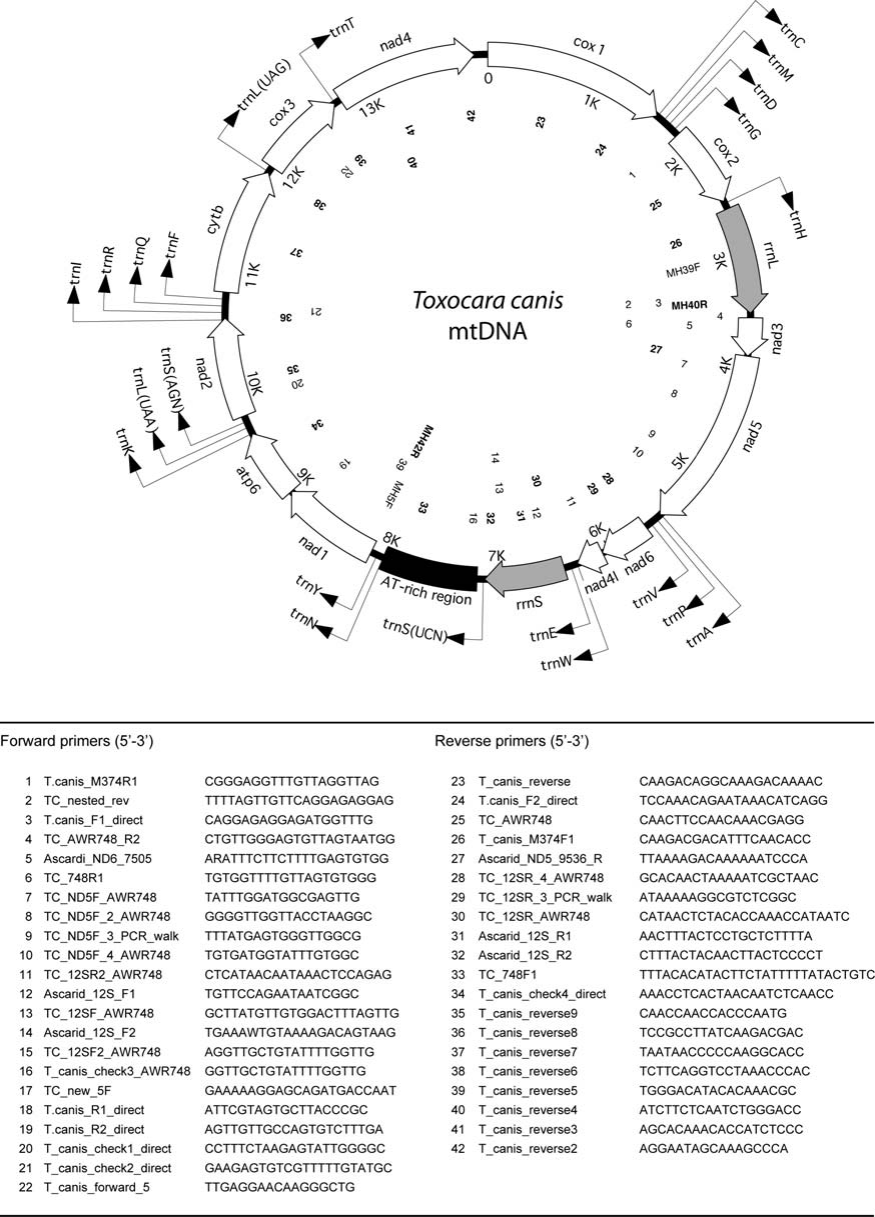
A map of the circular mitochondrial genome (mtDNA) of *Toxocara canis*. All 12 protein-coding genes and the large and small ribosomal subunits of the rRNA genes are indicated in italics. Each tRNA gene is identified by its anticodon (in brackets). The direction of transcription is indicated by an arrow. The positions of oligonucleotide primers (see table) used for PCR-amplification or sequencing are indicated in the map (drawn to scale).

Using each of the primer pairs MH39F-MH42R and MH5F-MH40R [30],[35],[36], two regions of the entire mitochondrial genome (of ∼5 and 10 kb, respectively) were amplified by the long PCR (Expand 20 kb^PLUS^ kit, Roche) from ∼20 ng of genomic DNA from sample Tcn2. The cycling conditions in a 2400 thermocycler (Perkin Elmer Cetus) were: 92°C, 2 min (initial denaturation); then 92°C, 10 s (denaturation); 50°C, 30 s (annealing); 60°C (∼5 kb region) or 68°C (∼10 kb region), 10 min (extension) for 10 cycles, followed by 92°C, 10 s; 50°C, 30 s; 68°C or 60°C, 10 min for 20 cycles, with an elongation of 10 s for each cycle, and a final extension at 68°C or 60°C for 7 min [30]. Each PCR yielded a single amplicon, detected by agarose gel electrophoresis [30]. Each amplicon was column-purified (PCR-Preps, Promega) and subjected to automated sequencing, either directly or following cloning (TOPO XL PCR cloning kit, Invitrogen, according to instructions provided), employing a “primer-walking” strategy [36] (see Figure 1). Sequencing was performed using BigDye terminator (v.3.1) in a 3730 DNA Analyser (Applied Biosystems). The sequences obtained were assembled manually, aligned with the mitochondrial genome sequence of *Ascaris suum* [18] using the program Clustal X [37], and the circular map was drawn using the program MacVector v.9.5 (http://www.macvector.com/index.html). Amino acid sequences, translation initiation and termination codons, codon usage, transfer RNA (tRNA or *trn*) secondary structures, rRNA secondary structures and non-coding regions were predicted using standard approaches [35]. The structure and organization of the mitochondrial genome of *T. canis* was then compared with those of the nematodes *Anisakis simplex* (GenBank accession number AY994157; ref. [17]), *Ascaris suum* (X53453; ref. [18]) (order Ascaridida); *Brugia malayi* (AF538716; ref. [38]), *Dirofilaria immitis* (AJ537512; ref. [39]) and *Onchocerca volvulus* (AF015193; ref. [40]) (order Spirurida); *Ancylostoma duodenale* (AJ417718; ref. [35]) and *Necator americanus* (AJ417719; ref. [35]) (order Strongylida).

## Results

### Features and Organization of the Mitochondrial Genome of *Toxocara canis*

The circular mitochondrial genome of *T. canis* (Figure 1) was 14162 bp in length (GenBank accession number EU730761) and contained 36 genes: 12 protein-coding genes (adenosine triphosphatase subunit 6 [*atp6*], the cytochrome *c* oxidase subunits 1, 2 and 3 [*cox1*–*cox3*], cytochrome b (*cytb*) and the nicotinamide dehydrogenase subunits 1–6 [*nad1*–*nad6* and *nad4l*]), 22 tRNA genes (two coding for leucine and two coding for serine) and the small [*rrn*S] and large [*rrn*L] subunits of rRNA. Each protein-coding gene had an open reading frame (ORF), and all genes were located on the same strand and transcribed in the same direction (5′ to 3′) (Figure 1), consistent with the mitochondrial genomes of other secernentean nematodes characterized to date [16]. The gene arrangement for the mitochondrial genome of *T. canis* was consistent with that of GA2 [41]. This gene arrangement has been reported previously for members of the Ascaridida, including *Anisakis simplex* [17] and *Ascaris suum* [18] as well as members of the order Strongylida, such as the hookworms *Ancylostoma duodenale* and *Necator americanus* [35] as well as the barber's pole worm, *Haemonchus contortus* [42]. However, consistent with the mitochondrial genomes characterized to date for other Ascaridida (but not the Strongylida), the AT-rich region for *T. canis* was located between *rrn*S and *nad1*, flanked (5′) by the genes *trn*S (UCN) and (3′) by *trn*N and *trn*Y.

### Nucleotide Contents and Codon Usage

The coding strand of the mitochondrial genome sequence of *T. canis* consisted of 21.6% A, 9.4% C, 22.1% G and 46.7% T (Table 1). Though AT-rich (68.4% AT), the sequence had a slightly lower AT content than has been reported for other nematode species (∼70–80%; refs. [17],[18],[35],[39],[41],[42]). In the protein-coding genes, the AT-contents varied from 63.1% (*cox1*) to 73.4% (*nad6*), with the overall ranking (increasing richness) of *cox1*, *cox3*, *nad1*, *cox2*, *cytb*, *nad4*, *atp6*, *nad5*, *nad2*, *nad4L*, *nad3* followed by *nad6*. To date, studies of secernentean nematodes have shown that the cytochrome *c* oxidase genes tend to have the lowest AT-contents [35],[39],[41],[42]. Although the overall AT-content of the mitochondrial genome sequence of *T. canis* was ∼2.8% and ∼3.6% less than those of *Anisakis simplex* (71.2%; ref. [17]) and *Ascaris suum* (72.0%; ref. [18]), respectively, there was no appreciable impact on the relative amino acid codon usage in the protein-coding genes. As has been reported for other secernentean nematodes (e.g., refs. [17],[18],[35],[39],[41],[42]), the usage in the protein-coding genes favoured codons with many A or T residues (e.g., 13.7% were TTT [phenylalanine]) over those with many C or G residues (e.g., none were CGA [arginine]) (data not shown).

**Table 1:** Lengths and A+T contents (%) of the sequences of the 12 protein-coding genes, the large and small ribosomal RNA genes, the AT-rich region and of the entire mitochondrial genome of *Toxocara canis*.

Mitochondrial gene/region	Length (bp)	A	C	G	T	AT
*atp6*	599	17.4	8.0	23.2	51.4	68.8
*cox1*	1575	18.0	11.3	25.4	45.1	63.1
*cox2*	698	20.9	9.9	24.5	44.7	65.6
*cox3*	768	16.7	10.6	24.2	48.6	65.2
*cytb*	1101	17.8	9.4	24.2	48.6	66.4
*nad1*	880	16.0	10.3	23.0	49.0	65.0
*nad2*	855	17.7	7.8	21.6	52.6	70.3
*nad3*	330	20.4	3.1	24.2	52.4	72.8
*nad4*	1230	19.3	21.8	10.5	48.5	67.7
*nad4L*	233	21.0	6.9	21.9	50.2	71.2
*nad5*	1578	19.5	8.8	21.9	49.9	69.4
*nad6*	435	18.6	8.7	17.7	54.9	73.6
*rrn*L	924	25.4	7.5	20.0	46.3	71.9
*rrn*S	693	30.3	10.7	22.4	36.2	66.5
AT-rich	828	38.6	9.9	11.8	39.5	78.1
Genome	14162	21.6	9.4	22.1	46.7	68.4

**Figure 2:**
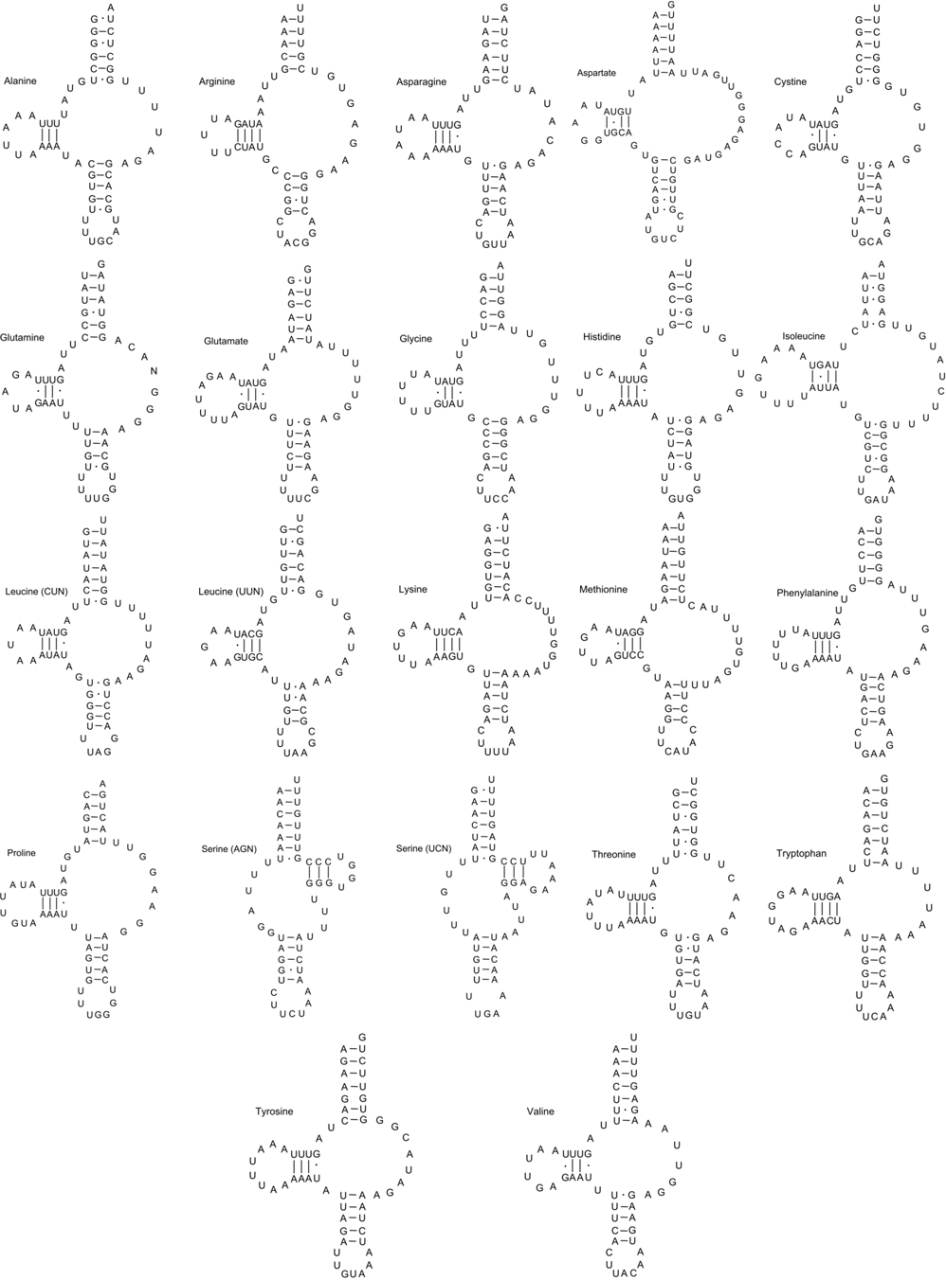
Secondary structures predicted for the 22 tRNA genes in the mitochondrial genome of *Toxocara canis* (cf. [15],[18]).

**Figure 3:**
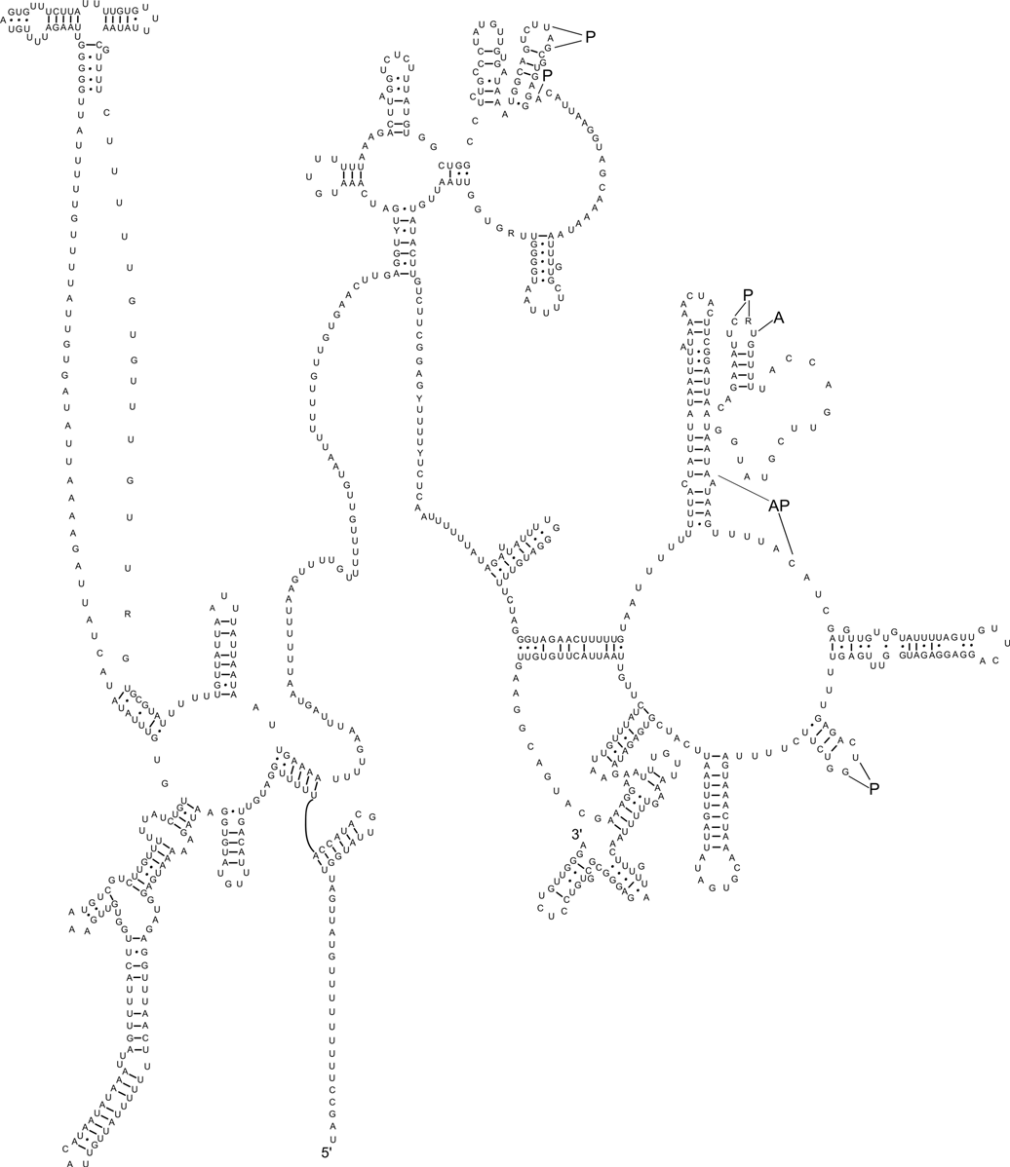
The secondary structure predicted for the large subunit (*rrn*L) of the rRNA gene in the mitochondrial genome of *Toxocara canis*. Bonds between C∶G and U∶A are indicated by a straight line and those between U∶G by a closed circle (cf. [35]). Binding sites for the amino-acyl trn (A), peptidyl-transferase (P) or both (AP) [101] are indicated by lines.

**Figure 4:**
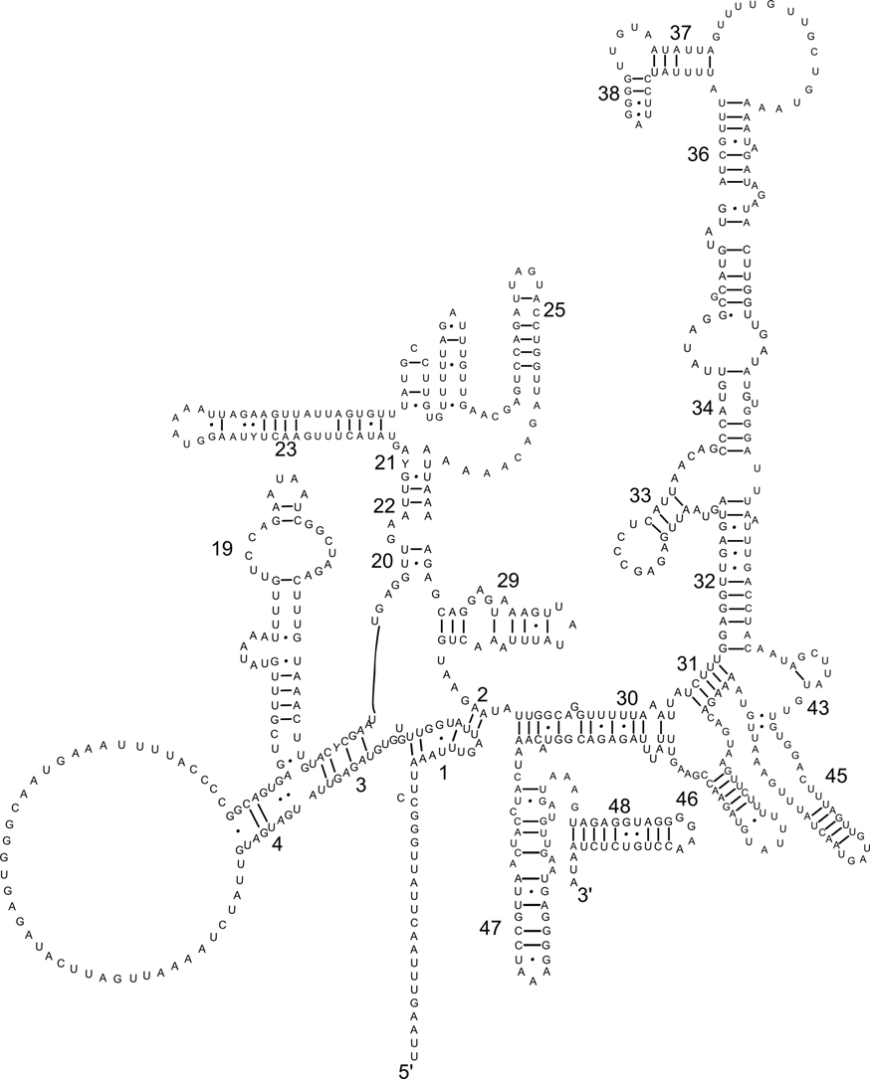
The secondary structure predicted for the small subunit (*rrn*S) of the rRNA gene in the mitochondrial genome of *Toxocara canis*. Bonds between C∶G and U∶A are indicated by a straight line and those between U∶G by a closed circle [35]. Conserved secondary structure elements [102] indicated by numbers 1–48.

All but the two serine tRNAs (AGN and UCN) had a predicted secondary structure containing a DHU arm and loop and a TV-replacement loop instead of the TψC arm and loop (Figure 2). As reported previously for secernentean nematodes [15],[16],[43],[44], the two serine tRNAs each contained the TψC arm and loop but lacked the DHU arm and loop. The *rrn*L and *rrn*S genes were 923 and 693 bp in length, respectively; the predicted secondary structure of each of these two genes are displayed in Figure 3 (*rrn*L) and Figure 4 (*rrn*S). The AT-content of the sequences of *rrn*L, *rrn*S and the AT-rich (“control”) region were 77.9%, 66.5% and 78.1%, respectively. The relatively low AT-richness exhibited in the mitochondrial genome of *T. canis* was pronounced for the rRNA genes: The AT-content of the *rrn*L sequence was 4.2% and 4.9% less compared with *Anisakis simplex* (76.1%) [17] and *Ascaris suum* (76.8%) [18], respectively. The AT-content of the *rrn*S sequence of *T. canis* was 5.5% and 5.4% less than that reported for *Anisakis simplex* (72.0%) [17] and *Ascaris suum* (71.9%) [18], respectively. Interestingly, the AT-content of the *T. canis* mitochondrial rRNA genes does not alter their predicted secondary structures with respect to those of other secernentean nematodes studied to date [35],[39],[41],[42].

**Figure 5:**
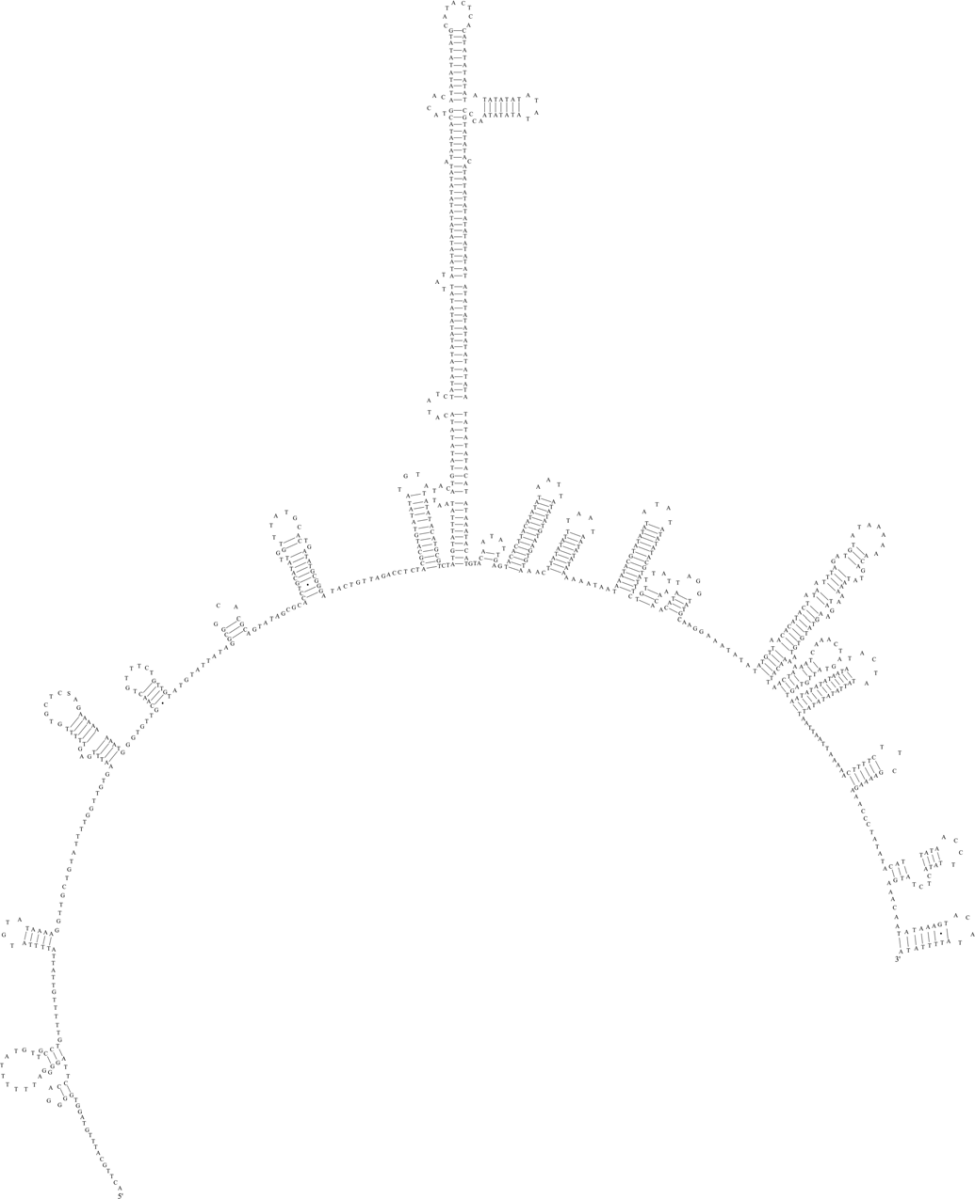
Secondary structure predicted for the AT-rich region in the mitochondrial genome of *Toxocara canis*.

The AT-rich region (Figure 1) was 828 bp in length and was predicted to exhibit a complex secondary structure (Figure 5). In addition, the AT-rich region contained 13 regions consisting of a varying numbers of the dinucleotide (AT) repeat (n = 3 to 21) between nucleotide positions 307 and 806 (see Figure 5). The presence of multiple AT repeats was similar to that described in the AT-rich region of several parasitic nematodes, including *Ascaris suum* [18], *Anisakis simplex* [17], *Ancylostoma duodenale* and *Necator americanus* [35], but distinct from the repetitive elements (CR1–CR6) within the AT-rich region of the free-living nematode *Caenorhabditis elegans* [18].

### Comparative Analysis with Other Nematodes

Pairwise comparisons were made among the amino acid sequences inferred from individual protein-coding genes and the nucleotide sequences of the rRNA genes in the *T. canis* mitochondrial genome with those representing seven other nematodes (of the orders Ascaridida, Spirurida and Strongylida) (Table 2). The amino acid sequence similarities in individual inferred proteins ranged from 70.3% (NAD2) to 94.4% (COX1) between *T. canis* and *Ascaris suum* and from 74.3% (CYTB) to 93.1% (COX1) between *T. canis* and *Anisakis simplex*. The amino acid sequence similarities between *T. canis* and each species of Spirurida (*Brugia malayi*, *Dirofilaria immitis* and *Onchocerca volvulus*) or Strongylida (*Ancylostoma duodenale* and *Necator americanus*) included ranged from 21.5% (ATP6) to 51.6% (COX1) and from 49.3% (NAD6) to 90.6% (COX1), respectively. The nucleotide sequence similarities (Table 2) in *rrn*S were 80.5–81.1%between *T. canis* and the two other species of Ascaridida, 59.0–60.0% between *T. canis* and the three members of the order Spirurida, and 71.8–72.4% between *T. canis* and the two species of Strongylida. In addition, the nucleotide sequence similarities in *rrn*L were 74.8–78.0%, 59.2–61.0% or 65.2–67.0% between *T. canis* and individual species representing the order Ascaridida, Spirurida or Strongylida, respectively (Table 2).

**Table 2:** Percentage of similarity in the amino acid sequences inferred from the 12 protein-coding genes and in the nucleotide sequence of each of the two ribosomal genes (*rrn*L and *rrn*S) upon pairwise comparison between *Toxocara canis* and seven other parasitic nematodes (representing the orders Ascaridida, Spirurida and Strongylida).

Protein/rRNA	*Asi*	*Asu*	*Bm*	*Di*	*Ov*	*Adu*	*Na*
ATP6	82.9	81.9	23.5	22.0	21.5	76.3	75.0
COX1	93.1	94.4	49.8	51.0	51.6	90.2	90.6
COX2	90.5	93.1	42.3	44.2	42.7	84.0	84.4
COX3	89.4	90.9	33.9	34.7	33.9	84.3	82.4
CYTB	74.3	80.5	49.4	48.3	49.5	73.5	71.8
NAD1	84.1	80.0	49.8	46.3	47.8	67.3	68.3
NAD2	74.7	70.3	36.7	33.3	35.2	51.0	51.4
NAD3	87.5	83.0	38.3	36.2	40.1	66.9	66.9
NAD4	81.9	82.3	46.6	45.9	47.0	62.3	61.8
NAD4L	87.1	88.3	35.4	38.7	43.7	70.1	71.4
NAD5	76.8	75.0	37.4	35.9	37.4	62.6	62.1
NAD6	77.0	76.3	29.8	28.0	28.6	49.3	57.6
*rrn*L	74.8	78.0	60.7	61.0	59.2	67.0	65.2
*rrn*S	80.5	81.1	59.2	60.0	59.0	72.4	71.8

*Asi* = *Anisakis simplex* (Ascaridida: Anisakidae) [17].

*Asu* = *Ascaris suum* (Ascaridida: Ascarididae) [18].

*Bm* = *Brugia malayi* (Spirurida: Onchocercidae) [38].

*Di* = *Dirofilaria immitis* (Spirurida: Onchocercidae) [39].

*Ov* = *Onchocerca volvulus* (Spirurida: Onchocercidae) [40].

*Adu* = *Ancylostoma duodenale* (Strongylida: Ancylostomatidae) [35].

*Na* = *Necator americanus* (Strongylida: Ancylostomatidae) [35].

## Discussion

### Implications for Testing the Phylogeny of Parasitic Nematodes

Pairwise comparisons of the amino acid sequences conceptually translated from the protein-coding genes as well as the nucleotide sequences of the ribosomal RNA genes indicated that the mitochondrial genome of *T. canis* most closely resembles those of selected members of the order Ascaridida. However, based on pairwise comparisons of sequence data, the next most similar nematode group is the Strongylida, but not the Spirurida. This finding is consistent with previous phylogenetic analyses of mitochondrial datasets, such as concatenated amino acid sequences for all 12 protein-coding genes [15] or gene arrangements [41]. These studies placed the Spirurida in a strongly supported clade separate from all other secernentean nematodes for which data were available, being in accordance with the evolutionary relationships based on traditional, taxonomic data [32], [45]–[47]. This placement of the Spirurida relative to the Ascaridida and Strongylida contrasts the classification of the Nematoda based on phylogenetic analysis of sequence data for the small subunit (SSU) of nuclear rRNA [48], inferring that members of the order Strongylida belong to “clade V”, whereas those of the orders Ascaridida and Spirurida are within “clade III”. The distinct taxonomic placement of the Spirurida relative to the Ascaridida and Strongylida is further evidenced by the variation in anti-codon usage in some of the mitochondrial tRNA genes of members of the Spirurida (i.e. *Brugia malayi*, *Dirofilaria immitis* and *Onchocerca volvulus*) as compared with the Strongylida and Ascaridida studied to date [15],[39]. The incongruence in the inferred relationships of these three nematode orders (i.e. among the Ascaridida, Strongylida and Spirurida) challenges the proposed molecular phylogeny for the Nematoda based on SSU sequence data [48] and stimulates further investigation of a broader range of nematodes.

### Implications for Systematic, Population Genetic, Epidemiological and Ecological Studies

There is major significance in the use of mitochondrial DNA markers for investigating the genetic make-up of species of the *Toxocara*, particularly given that there are no morphological features which allow the specific identification of some stages (e.g., larvae) [29] and given that cryptic species have been detected within the Ascaridoidea [27], [49]–[62]. In nematodes, mitochondrial DNA is proposed to be maternally inherited (cf. [16]) and is usually more variable in sequence within a species than nuclear ribosomal DNA [9]. Various different mitochondrial gene regions are suited to studying the population genetics of parasitic nematodes [8], [10], [15], [63]–[67]. However, surprisingly, there has been a paucity of information on the mitochondrial genomes of ascaridoid nematodes [16], which appeared to have related mainly to technical limitations and the cost associated with mitochondrial genome sequencing. To overcome this constraint, Hu et al. [30],[36] developed the long PCR approach applied herein to *T. canis*, which has broad applicability to a range of ascaridoids, including other species of *Toxocara*, *Toxascaris*, *Baylisascaris*, *Lagochilascaris* and members of the *Anisakis* complex [29],[68].

The characterization of the first complete mitochondrial genome sequence for *T. canis*, in the present study, provides a foundation for addressing ecological and epidemiological questions regarding this and related species. Conserved primers can be rationally and selectively designed to relatively conserved regions flanking “variable tracts” in the mitochondrial genome considered to be most informative (based on sequencing from a small number of individuals from particular populations, or genetic variants detected using nuclear ribosomal markers; refs. [29],[67]). Using such primers, single-strand conformation polymorphism (SSCP) analysis [69] can be applied to pre-screen large numbers of individuals representing different populations and, based on the ‘pre-screen’, samples representing the entire spectrum of haplotypic variability can be selected for subsequent sequencing and analyses. Such an approach has been applied effectively, for example, to explore the genetic make-up of the *Ascaris* populations in humans and pigs in six provinces in China [70]. This study indicated restricted gene flow between human *Ascaris* and porcine *Ascaris*, and supported the conclusions from other previous epidemiological and experimental investigations [71],[72] that pigs are not a significant source of *Ascaris* infection to humans in endemic regions.

Utilizing a range of mitochondrial gene markers (with differing degrees of intraspecific variability), such a mutation scanning-targeted approach is also readily and directly applicable to species of *Toxocara*. This is particularly relevant now, given that population variation and cryptic species have been detected within *Toxocara* [27],[28],[31] and that almost nothing is known about the transmissibility of these or other, as yet undetected, variants and/or cryptic species to humans and other hosts. For instance, early reports from Malaysia [25],[26] described the occurrence of a parasite in cats, which was identified as *T. canis*, based on the presence of an oesophageal ventriculus and spear-shaped cervical alae in the adult [73]. This parasite differed from the common species known to parasitize cats [73]–[76], such as *Toxocara cati*, which has arrow-shaped cervical alae, and *Toxascaris leonina*, which lacks a ventriculus. Because *T. canis* has been found only rarely in cats elsewhere in the world [76]–[81], the question arose as to the specific identity of this parasite in Malaysian cats. A molecular study, using markers in the first and second internal transcribed spacers (ITS-1 and ITS-2, respectively) of nuclear ribosomal DNA markers, was undertaken to genetically characterize specimens of this parasite, then called *Toxocara* sp. cf. *canis* [27]. The molecular investigation indicated clearly that *Toxocara* sp. cf. *canis* from Malaysian cats was genetically distinct from *T. canis* and *T. cati*, a conclusion which was supported by a subsequent morphological study of a number of ascaridoids from Malaysia [28]. Three morphological features (for lips, alae and spicules) were identified which consistently differentiated *Toxocara* sp. cf. *canis* from *T. canis*, *T. cati* and other congeners, such as *T. tanuki* (from canids), *T. apodemi* and *T. mackerrasae* (from rodents), *T. paradoxura* and *T. sprenti* (from viverrids), *T. vajrasthirae* (from mustelids) and *T. pteropodis* (from bats). Hence, the findings from the molecular and classical systematic studies supported the conclusion that *Toxocara* sp. cf. *canis* represented a distinct species, subsequently named *T. malaysiensis* [28].

Although *T. canis* is well recognized as the causative agent of toxocariasis in humans, including ocular larva migrans (OLM) and/or visceral larva migrans (VLM), other congeners, such as *T. malaysiensis*, *T. cati* and *T. vitulorum*, may have greater zoonotic importance than assumed [82],[83]. *T. malaysiensis* is of particular interest as a potential zoonotic pathogen, given its high prevalence (11%) in cats [26]. The transmissibility of this species to other host species (e.g., mouse, rat, rabbit and pig) warrants assessment, together with epidemiological surveys utilizing molecular tools employing genetic markers from the mitochondrial genome of *T. canis* as well as specific nuclear markers in the ITS-1 and/or ITS-2. The discovery of *T. malaysiensis* in cats in Malaysia [27] also raises important questions as to the identity and zoonotic potential of ascaridoids considered to represent *T. canis* in cats in other geographical regions, including South Africa, Panama, the USA and Czech Republic [76]–[81],[84], which provides a stimulus for the genetic characterization of additional *Toxocara* isolates from a broad range of hosts and geographical origins and to subsequently evaluate their potential to infect humans and/or other hosts.

From epidemiological and ecological perspectives, it would be interesting, utilizing mitochondrial genomic data, to confirm or refute the involvement of *Toxocara* in human VLM cases in Japan, currently considered to be caused by *Ascaris suum* based on serological evidence [85],[86], as there has been considerable controversy as to the specific identity of the causative agent of the disease in these instances [87]. It would also be particularly relevant to explore whether specific genotypes/haplotypes of *Toxocara canis* have a particular affinity to the human host and/or predilection sites in tissues to cause different types of toxocariasis and whether there are specific subpopulations of *T. canis* that undergo arrested development in tissues. Using molecular tools, in combination with traditional parasitological and serological methods, it should also be possible to characterize in detail experimental infections in “model host systems” (e.g., mouse, rabbit or pig) [24], [88]–[93]. Furthermore, mitochondrial markers would be useful for exploring the zoonotic risk of paratenic hosts, particularly those commonly encountered in an agricultural setting (e.g. chickens, ducks or pigs [94]–[98]), and determining the specific identity of eggs in the environment [99].

In conclusion, the present study emphasizes the relevance of the mitochondrial genome of *T. canis* defined herein, should provide a foundation for a range of systematic, population genetic, epidemiological, ecological and biological studies. Although the PCR-based sequencing-cloning approach used herein was effective, the PCR-coupled 454 technology platform [100], constructed recently for the direct sequencing of mitochondrial genomes from single nematodes [42], provides perhaps the most exciting development for large-scale, high throughput population genetic and mitochondrial genomic studies of nematodes and other organisms.
